# The Application of Non-Coding RNAs as Biomarkers, Therapies, and Novel Vaccines in Diseases

**DOI:** 10.3390/ijms26073055

**Published:** 2025-03-26

**Authors:** Lu-Xuan Yang, Hui Li, Zhi-Hui Cheng, He-Yue Sun, Jie-Ping Huang, Zhi-Peng Li, Xin-Xin Li, Zhi-Gang Hu, Jian Wang

**Affiliations:** 1 Guangxi Key Laboratory of Animal Breeding, Disease Control and Prevention, College of Animal Science and Technology, Guangxi University, Nanning 530004, China; luxuanyang@st.gxu.edu.cn (L.-X.Y.); huili@gxu.edu.cn (H.L.); zhihuicheng@st.gxu.edu.cn (Z.-H.C.); genuine749@163.com (H.-Y.S.); huangiieping@gxu.edu.cn (J.-P.H.); zp.li@gxu.edu.cn (Z.-P.L.); 2 Institute of Scientific Research, Guangxi University, Nanning 530004, China; kjclxx@gxu.edu.cn; 3 College of Animal Science and Technology, Northwest A&F University, Yangling 712100, China

**Keywords:** ncRNAs, biomarker, therapy, vaccine

## Abstract

Non-coding RNAs (ncRNAs) are a class of RNAs that largely lack the capacity to encode proteins. They have garnered significant attention due to their central regulatory functions across numerous cellular and physiological processes at transcriptional, post-transcriptional, and translational levels. Over the past decade, ncRNA-based therapies have gained considerable attention in the diagnosis, treatment, and prevention of diseases, and many studies have revealed a significant relationship between ncRNAs and diseases. At the same time, due to their tissue specificity, an increasing number of projects have focused on the application of ncRNAs as biomarkers in diseases, as well as the design and development of novel ncRNA-based vaccines and therapies for clinical use. These ncRNAs may also drive research into the potential molecular mechanisms and complex pathogenesis of related diseases. However, new biomarkers need to be validated for their clinical effectiveness. Additionally, to produce safe and stable RNA products, factors such as purity, precise dosage, and effective delivery methods must be ensured to achieve optimal bioactivity. These challenges remain key issues in the clinical application of ncRNAs. This review summarizes the prospects of ncRNAs as potential biomarkers, as well as the current research status and clinical applications of ncRNAs in therapies and vaccines, and discusses the challenges and expectations of ncRNAs in disease diagnosis and drug therapy.

## 1. Introduction

In the past few years, with the advancement and progress of deep RNA sequencing technology, research on RNAs has gone beyond just mRNA, which encodes proteins. Meanwhile, non-coding RNAs (ncRNAs) have received increased attention and investigation due to their diverse roles in multiple biological activities, such as cell survival [1], immune response [2], disease development, and so on [3]. Typically, ncRNAs are classified into two major categories based on their functions: “Housekeeping ncRNAs”, which are widely expressed within cells and primarily regulate basic cellular functions that are essential for cell survival. These ncRNAs maintain relatively stable levels and encompass transfer RNAs (tRNAs), ribosomal RNAs (rRNAs), small nuclear RNAs (snRNAs), small nucleolar RNAs (snoRNAs), and telomerase RNAs (TERCs). The other class of ncRNAs possesses regulatory functions, capable of modulating gene expression at both the transcriptional and post-transcriptional levels. This category includes microRNAs (miRNAs), circular RNAs (circRNAs), long non-coding RNAs (lncRNAs), small interfering RNAs (siRNAs), and PIWI-interacting RNAs (piRNAs). These ncRNAs have different structures and functions, which serve as the basis for their classification (Table 1).

There is an inseparable relationship between ncRNAs and diseases. In addition to influencing the occurrence and development of diseases, many of these regulatory ncRNAs have also been described as biomarkers that can be used to predict and diagnose various diseases, including cancers [4], cardiovascular diseases [3], and neurological disorders [3,5]. Their emergence provides more non-invasive and specific options for disease diagnosis. In therapeutics, an increasing number of valuable studies have shown that ncRNAs can regulate gene expression networks, providing new directions for targeted therapy. Some ncRNA-based therapies have been applied to cancer, cardiovascular diseases, genetic disorders, and metabolic diseases [6]. Additionally, in recent years, circRNA vaccines have shown great potential in infectious disease prevention and immunotherapy. They are more stable than mRNA vaccines and can induce longer-lasting immune responses [7]. Although ncRNAs have been increasingly applied in diseases, their clinical translation still faces many challenges. For example, as a biomarker, the threshold of expression levels needs to be considered; as a drug, safety and efficacy must be evaluated; and as a vaccine, synthesis and delivery require careful consideration. Based on recent studies, this review primarily discusses the research on ncRNAs as potential biomarkers in diseases and their breakthroughs and applications in novel vaccines and therapies, as well as the challenges they face and future prospects, which aims to provide new insights into the research related to the diagnosis, treatment, and prevention of diseases using ncRNAs.

**Table 1:** Common types of ncRNAs and their descriptions.

Types of RNA	Features and Introduction	References
tRNAs	These possess a cloverleaf structure, deliver the correct amino acids to the ribosome, and facilitate the formation of polypeptide chains	[8,9]
rRNAs	The structural components of the ribosome	[10]
snRNAs	A class of small RNA molecules in the nucleus of eukaryotic cells that are capable of processing pre-mRNAs	[11]
snoRNAs	Regulatory factors promoting rRNA maturation	[12]
TERCs	The RNA component of telomerase, which provides a structural scaffold for the assembly of the telomerase complex	[13,14]
miRNAs	With a length of approximately 19~24 nucleotides (nts), these serve as post-transcriptional regulatory factors of gene expression	[15]
circRNAs	These lack free ends and have covalently closed loop structures, and can function as miRNA sponges, RNA-binding protein sponges, and translation regulatory factors	[16,17]
lncRNAs	With a length exceeding 200 nts, these mediate the post-transcriptional control of signal transduction pathways, translation processes, and gene expression	[18]
siRNAs	These are primarily involved in RNA interference to regulate gene expression	[19]
piRNAs	These have diverse functions including gene regulation, transposon suppression, epigenetic programming, and antiviral defense, among others	[20,21]

## 2. NcRNAs as Diagnostic Biomarkers

NcRNAs serve as important potential biomarkers in the diagnosis and prognosis of diseases. They exhibit stability and universality, being expressed in various tissues, blood, urine, and saliva [22,23,24], and also possess certain disease specificity [25,26], which makes them highly suitable as biomarkers for disease diagnosis, prognosis, and treatment. In current research on ncRNAs, studies on miRNAs are the most extensive, while research on circRNAs and lncRNAs is also progressing further. Therefore, this section discusses the applications of miRNAs, circRNAs, and lncRNAs in diseases.

### 2.1. MiRNAs as Biomarkers

MiRNAs are the most abundant small RNAs [27], playing crucial roles in cell differentiation [28,29], apoptosis [30,31], organ development [32], metabolism [33,34], and others. They regulate target gene expression by binding to the 3′ untranslated region (3′UTR) of mRNA, leading to translation inhibition or degradation [35]. Tissue-specific miRNAs are often associated with diseases related to specific tissues, and changes in their expression patterns have been confirmed in various human diseases, including cancer, cardiovascular diseases, neurological disorders, genetic diseases, metabolic disorders, and viral pathogenesis. Moreover, the dysregulation of miRNAs has been proven to be a pathogenic factor in disease progression [36].

The differential expression of certain miRNAs in various stages of different diseases has led to their continued investigation as potential biomarkers. For example, miR-21 and miR-22 are two highly frequent miRNAs in cancer and are abnormally expressed in various types of cancer [37,38]. In a study, by comparing the expression levels of miR-21 and miR-22 in the serum of 80 epithelial ovarian cancer (EOC) patients and 80 healthy volunteers, researchers found that these two miRNAs exhibited different numerical changes in different stages of epithelial EOC. In the late stage of EOC, the fold change in miR-21 expression was higher than in the early stage, while miR-22 exhibited the opposite trend [39]. This opposite trend allows these two miRNAs to serve as diagnostic biomarkers in the early stages of EOC and enables the prediction of ovarian cancer progression over time by monitoring their fold change values. Moreover, in recent research, Duan et al. [40] discovered through sequencing that the expression levels of miR-92a-3p, miR-425-5p, and miR-185-5p were significantly elevated in the urine of patients with IgA nephropathy (IgAN). Gene Ontology (GO), the Kyoto Encyclopedia of Genes and Genomes (KEGG), and subsequent dual-luciferase reporter assays confirmed that in renal tubular epithelial cells, the target gene of miR-185-5p is tight junction protein 1 (*TJP1*). In vitro experiments also indicated that miR-185-5p mimics can reduce the expression of TJP1 in human kidney 2 (HK-2) cells and promote the transition of renal tubular epithelial cells to a fibrotic phenotype. During the progression of IgAN, tubulointerstitial fibrosis is an important histological marker [41]. This study provides strong evidence that the expression levels of miR-92a-3p, miR-425-5p, and miR-185-5p in urine can serve as biomarkers for IgAN. This non-invasive detection method is undoubtedly simpler and faster, aiding in early diagnosis. Additionally, miRNAs can also serve as biomarkers for liver injury and rejection after liver transplantation [42]. Plasma miRNAs, including miR-181a-5p, miR-155-5p, and miR-122-5p, are significantly upregulated in patients with acute T-cell-mediated rejection (TCMAR) and subclinical rejection (SCR) after liver transplantation. Before transplantation, the expression levels of miR-155-5p and miR-181a-5p exhibit different trends between the two groups: miR-155-5p is significantly higher in TCMAR patients than in SCR patients, whereas miR-181a-5p is significantly higher in SCR patients than in TCMAR patients. This difference provides valuable insights for diagnosis and prognosis before and after liver transplantation, making these miRNAs potential candidates for a biomarker panel in liver injury following transplantation [43].

### 2.2. CircRNAs as Biomarkers

CircRNAs are covalently closed loops formed by single-stranded RNA without free ends. The majority of circRNAs originate from exons and are highly expressed in organisms. Their circular and distinct structure renders circRNAs more stable and less prone to degradation by RNA exonucleases when compared to linear RNAs [44]. This high stability provides favorable conditions for their use as biomarkers. CircRNAs serve multiple functions; they can act as competitive endogenous RNAs or miRNA sponges [45], and they can also interact with RNA-binding proteins [46]. Furthermore, some circRNAs have the capability to undergo translation to produce proteins [47] and regulate gene transcription [48]. Similarly, circRNAs exhibit differential expression in various diseases, making them potential biomarkers or therapeutic targets for these conditions [25].

The oral glucose tolerance test (OGTT) and overnight fasting plasma glucose (FPG) have long been the primary methods for detecting diabetes [49]. However, these methods involve many uncertainties, are time-consuming, and are complex. Using specific circRNAs in the blood as biomarkers allows for lower-cost, more convenient sampling and detection, enabling a faster determination of disease progression. For example, Zhao et al. [50] extracted whole blood from three groups: healthy individuals, individuals with prediabetes, and those with type 2 diabetes (T2DM). RNA was extracted and the relative expression levels of circRNAs were measured using q-PCR. The results showed that the expression levels of hsa_circ_0054633 and hsa_circ_0068087 were significantly different among the three groups and increased sequentially in the healthy control group, prediabetes group, and T2DM group. They then used a microarray analysis comparing the expression of circRNAs in the peripheral blood of six healthy individuals and six T2DM patients. They found that hsa_circ_0054633 had the largest area under the curve (AUC) and a lower *p*-value, indicating its potential diagnostic capability for prediabetes and T2DM. Additionally, pancreatic ductal adenocarcinoma (PDAC) is a cancer with a high mortality rate [51]. Patients often do not exhibit prominent symptoms clinically, and its diagnosis is usually made incidentally during routine imaging. This often leads to many patients being diagnosed in an advanced stage or after distant metastasis has occurred [52]. Recently, Xu et al. [53] developed a circular circRNA biomarker panel for the identification of PDAC. They identified five circRNAs in plasma for the active detection of PDAC and combined the circRNA-based panel with the serum tumor marker, cancer antigen 19-9 (CA19-9) glycoprotein, which enhances the diagnostic performance for early-stage PDAC. Additionally, circRNAs are widely involved in the regulation of bone metabolism [54]. Hsa_circ_0006859 is one of the most significantly upregulated circRNAs in serum exosomes of postmenopausal osteoporosis patients. It can promote the expression of ROCK1 through the *miR-431-5p/ROCK1* axis, thereby inhibiting osteoblast differentiation and promoting adipogenesis. These results suggest that Hsa_circ_0006859 is a potential biomarker for postmenopausal osteoporosis and provide a direction for the targeted therapy of related diseases [55].

### 2.3. LncRNAs as Biomarkers

LncRNAs are defined by their length, which exceeds 200 nucleotides. Based on their relative position to coding genes on the chromosome, lncRNAs can be broadly categorized into five classes: antisense, sense, intronic, intergenic, and bidirectional. They are primarily transcribed by RNA polymerase II and lack apparent open reading frames [56]. LncRNAs have various functions based on their subcellular localization, including gene imprinting [57], histone modification [58], chromatin remodeling [58], transcriptional activation [59], transcriptional interference [60], and cell cycle regulation [61].

LncRNAs can also serve as potential biomarkers. For example, PCA3 is a well-known prostate cancer-specific lncRNA [62]. It is overexpressed in prostate cancer (PCa) but not associated with other prostate conditions [63,64]. This makes PCA3 a suitable potential biomarker for PCa. Previously, serum prostate-specific antigen (PSA) was an important indicator for prostate cancer diagnosis, while the histopathological evaluation of prostate biopsy was the decisive factor for confir mation [65]. PCA3 can be collected from urine and plasma, which undoubtedly reduces the difficulty of sample collection and shortens the testing time. Additionally, inflammatory bowel disease (IBD) is a chronic, relapsing condition that includes Crohn’s disease (CD) and ulcerative colitis (UC) [66]. Heydari et al. [67] compared five previously reported lncRNAs related to IBD (including H19 [68], CDKN2B-AS1 [69], TUG1 [70], GAS5 [71], and CRNDE [72]). By analyzing the expression of each lncRNA in patient tissue samples and plasma extracellular vesicles (EVs), they found that the expression level of H19 was significantly higher in IBD patients than in the healthy control group. Additionally, compared to rheumatoid arthritis (RA) and irritable bowel syndrome (IBS) patients, H19 expression in plasma EVs was significantly elevated, confirming that H19 can serve as a potential biomarker for IBD with strong discriminatory ability. In addition, lnc-GAS5 is involved in the development of atherosclerosis and coronary heart disease (CHD), with miR-21 as its target [73]. Jiang et al. [74] detected the expression levels of lnc-GAS5 and miR-21 in CHD patients and the control group using RT-qPCR. They found that the expression level of lnc-GAS5 was significantly increased in CHD patients, while miR-21 showed an opposite trend, with a negative correlation between the two in CHD patients. Additionally, lnc-GAS5 was positively correlated with the Gensini score, which reflects the severity of coronary artery stenosis. These results suggest that lnc-GAS5 and its target, miR-21, could serve as potential biomarkers for CHD.

Some examples of ncRNAs with the potential to be used as disease biomarkers are summarized in Table 2.

## 3. NcRNAs in Target Therapy

The ability of ncRNAs to regulate the expression of target genes represents the great potential for target therapies. Currently, various RNA-based therapies have been developed, including antisense oligonucleotides (ASOs), short hairpin RNAs (shRNAs), small interfering RNAs (siRNAs), anti-microRNAs (anti-miRNAs), aptamers, and others [6]. In 1998, Fomivirsen, the world’s first antisense oligonucleotide-based medicine was approved by the United States Food and Drug Administration (FDA) and the European Medicines Agency (EMA) to treat cytomegalovirus (CMV) retinitis in patients with acquired immunodeficiency syndrome (AIDS) [104]. This drug consists of 21 thio-oligonucleotides with the nucleotide sequence 5′-GCGTTTGCTCTTCTTCTTGCG-3′, which can complement the mRNA of the major immediate-early region proteins of human cytomegalovirus, and it has an approximate half-life of about 55 h in the human body, making it an effective antiviral medication targeting CMV retinitis [105]. Since then, an increasing number of drugs based on ncRNAs have been approved and utilized. In 2018, Patisiran became the first siRNA drug to be approved by the FDA. It reduces mutant and wild-type transthyretin protein production by targeting the 3′ untranslated region of transthyretin mRNA for the treatment of hereditary transthyretin-mediated amyloidosis [106].

In addition, there are other ncRNA therapies under exploration, and different ncRNAs have shown varying effects in clinical trials. miRNA-based therapies offer several advantages. On one hand, miRNAs naturally exist in the human body, and they have established mechanisms and can selectively target downstream targets [107]. On the other hand, they can affect multiple genes within the same pathway, leading to broader yet specific responses [108]. Previously, the Kasinski team published a study where they developed a novel cancer therapy using a modified form of miR-34a to halt cancer cell division. In preclinical trials with mice, treatment using fully modified miR-34a targeting tumors resulted in the cessation of tumor growth within a 21-day assessment period, with some animals achieving complete remission. By contrast, untreated tumors doubled in size during the same period. These findings have the potential to rejuvenate miR-34a as an anticancer agent and provide robust support for clinical trials [109].

In another study, Liu et al. [110] identified a lncRNA enriched in cardiomyocytes, named LncHrt. Knocking out LncHrt impairs cardiac homeostasis, whereas overexpressing it using an AAV9 vector protects the heart from myocardial infarction and rescues the transcriptome in infarcted hearts. This indicates the therapeutic potential of LncHrt in myocardial infarction, providing a theoretical foundation for LncHrt as a novel treatment for ischemic heart disease.

Table 3 lists current RNA therapies currently approved by the FDA and/or EMA.

## 4. NcRNAs in Novel Vaccines

The RNA vaccines have the advantages of high efficacy, strong adaptability, and good tolerability [128]. For example, mRNA vaccines can directly encode antigens after entering the cytoplasm, characterized by their non-integrating, non-infectious nature and good tolerability [129,130]. However, mRNA vaccines are highly unstable in terms of storage and intracellular delivery, prone to degradation by nucleases, which significantly increase the risks and production costs associated with mRNA vaccines. Therefore, seeking stable alternative types of RNA vaccines to replace them is a new research direction.

Currently, breakthrough progress has been made in vaccine research based on ncRNAs, with the focus primarily on circRNAs. This is because circRNA-based vaccines are mainly derived from linear RNA precursors (pre-circRNAs) that are cyclized in vitro and then rely on the host organism’s translation system to generate antigens, thereby stimulating immune responses [131]. Compared to linear mRNAs, circRNAs have a covalently closed loop structure, which makes them highly stable and resistant to degradation by exonuclease-mediated cleavage [7]. Additionally, circRNAs exhibit lower immunogenicity and cytotoxicity, thereby reducing side effects [132]. Furthermore, circRNAs can elicit more durable immune responses [132,133]. These advantages demonstrate the immense potential of circRNAs in vaccine research.

**Figure 1:**
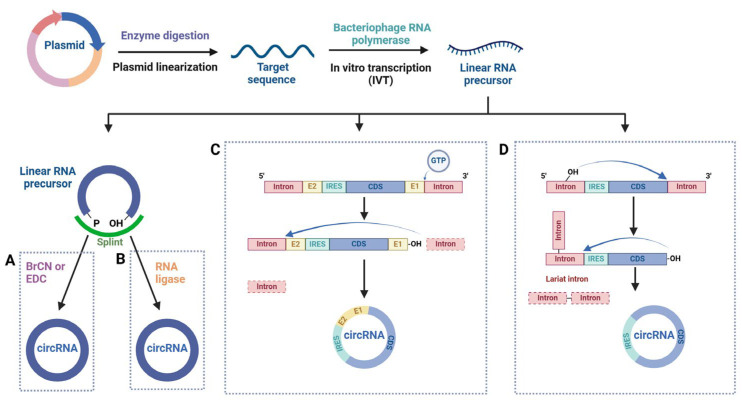
In vitro synthesis methods of circRNAs. (**A**) The chemical strategy. (**B**) The enzymatic strategy. (**C**) Group I intron-based permuted intron–exon (PIE) system. The sequence to be circularized is inserted into the exon regions (E1 and E2). In the presence of guanosine triphosphate (GTP), self-splicing is initiated, resulting in circularization, and two half-intron fragments are released. The circularized circRNA contains E1 and E2 (referred to as the scar sequence), which may affect the target sequence. (**D**) Group II intron-based PIE system. This system has no redundant sequences. After the lariat intron is removed, the ends of the exon are joined to form a circle.

The synthesis of linear RNA precursors is typically carried out using the in vitro transcription (IVT) method. In this process, plasmid DNA is used as the template, and bacteriophage RNA polymerase is employed to generate large numbers of RNA molecules. Subsequently, chemical, enzymatic, or ribozyme-mediated methods are used to circularize the linear RNA precursors [134,135,136]. The chemical strategy most commonly employs condensation reagents such as hydrogen bromide (BrCN) or 1-ethyl-3-(3-dimethylaminopropyl) carbodiimide (EDC) to ligate two RNA strands with terminal 5′-phosphate and 3′-hydroxyl groups [137,138]. The enzymatic strategy typically uses T4 DNA ligase (T4 Dnl), T4 RNA ligase 1 (T4 Rnl1), or T4 RNA ligase 2 (T4 Rnl2) to catalyze the ligation of RNA ends with 5′-phosphate and 3′-hydroxyl groups [136]. Ribozyme-mediated ligation is the most common approach, with Group I and Group II introns exhibiting ribozyme RNase activity to connect linear RNA precursors into circRNA through self-catalyzed intron splicing reactions [131,139,140]. The methods for in vitro synthesis of circRNA are shown in Figure 1.

**Figure 2:**
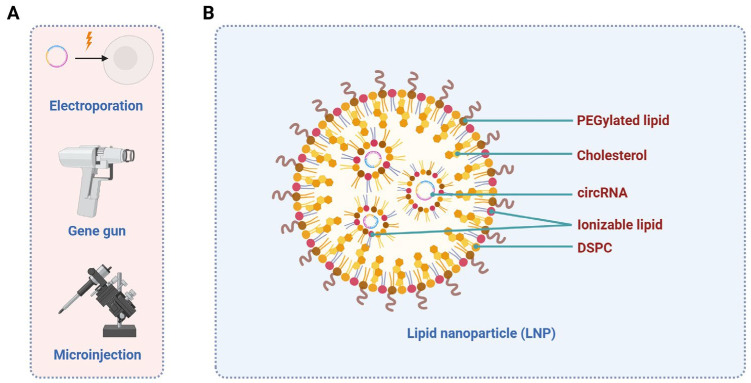
Delivery methods of circRNA vaccines. (**A**) Early experimental delivery methods include electroporation, gene gun, and microinjection. (**B**) The structure of using LNP to deliver circRNA vaccines.

The successful delivery of circRNAs is crucial in vaccination. CircRNAs are typically large molecules, which makes it difficult for them to diffuse in and out of cells. In the delivery of mRNA vaccines, electroporation was one of the earliest methods [141], and techniques such as gene gun [142] and microinjection [143] can also deliver mRNA antigens. Similarly, these methods can also be applied to the delivery of circRNA vaccines. With the advancement of delivery strategies, lipid nanoparticles (LNPs), including solid lipid nanoparticles (SLNs) and nanostructured lipid carriers (NLCs), have become the most widely used. They exhibit more complex structures, enhanced physical stability, easier synthesis, and greater loading capacity [140,144,145]. Previous delivery methods and the structure of LNP are shown in Figure 2.

Research on circRNA vaccines is gradually innovating and advancing. Previous studies have shown that tumor-specific antigens (TSAs) primarily originate from non-coding regions, and that the non-coding genome may encode peptides that bind to human leukocyte antigens (HLAs) as cryptic antigens to stimulate adaptive immunity [146,147]. Building on this basis, Huang et al. [148] compared the mass spectrometry analysis of the HLA class I (HLA-I) peptidome in human breast cancer samples with ribosome sequencing. They confirmed that circFAM53B can undergo non-canonical translation as a tumor-specific circRNA to produce cryptic antigenic peptides that bind to HLA-I. These cryptic peptides can initiate naive CD4 and CD8 T cells in an antigen-specific manner, thereby inducing an anti-tumor immune response. This suggests that using tumor-specific circRNA as a vaccine against malignant tumors holds considerable therapeutic potential.

In addition, synthetic circRNA vaccines have found more applications. For instance, during the global coronavirus disease 2019 (COVID-19) pandemic, Qu et al. [149] utilized an IVT strategy to generate SARS-CoV-2 circRNA^RBD^, which can express the trimeric receptor-binding domain (RBD) of the spike protein. They validated its ability to induce sustained immune responses and neutralize antibodies in mice and rhesus monkeys.

Li et al. [150] developed a lipid nanoparticle (LNP) system for both the in vitro and in vivo delivery of circRNAs to study the functionality of circRNA vaccines. They prepared circRNA^OVA-luc^-LNP (OVA [257-264]-luciferase-coding circRNA) vaccines for subsequent research. The results showed that circRNA-LNPs elicited effective innate immune responses and significant antigen-specific responses, demonstrating good anti-tumor effects in various mouse tumor models.

Research on circRNA vaccines is still developing, and their use in humans still requires a lot of clinical practice. Table 4 summarizes some circRNA vaccines that have been developed or are in development.

## 5. Discussions: Challenges and Opportunities

Although an increasing number of studies have demonstrated the great potential of ncRNAs in biomarkers, therapeutic targets, and novel vaccines, their transition from clinical trials to widespread application still faces significant challenges.

Discovering a new ncRNA biomarker can undoubtedly bring great convenience to disease diagnosis and prognosis, but identifying a new biomarker is not an easy task. Firstly, the threshold value of ncRNAs as biomarkers is a crucial indicator. Establishing a clear threshold for disease diagnosis requires a large number of experimental samples. At the same time, factors such as age differences, gender differences, and regional variations also need to be considered. Secondly, there are currently very few single biomarkers that can truly be used for disease diagnosis, and some ncRNAs exhibit similar expression patterns across different diseases [159], making them less suitable as specific disease markers. By contrast, combinational biomarkers can provide higher sensitivity and specificity. For example, in previous reports, although PCA3 has been approved as a PCa diagnostic marker, it has certain limitations, such as low sensitivity in patients with low-grade prostate cancer. Studies have shown that the elevated expression of the ERG oncogene in prostate cancer patients is due to its fusion with transmembrane protease serine 2 (TMPRSS2) [160]. Combining PCA3 with the *TMPRSS2-ERG* gene fusion for joint analysis can further improve diagnostic accuracy [161,162].

Additionally, the development and application of ncRNA therapies face numerous challenges. In the same disease, different types of ncRNAs may exhibit abnormal expression. Investigating disease pathogenesis and identifying specific ncRNAs require extensive time for exploration. Meanwhile, ensuring that the therapy targets only the intended ncRNAs without affecting other ncRNAs or gene expression remains a significant challenge. Furthermore, factors such as maintaining an effective drug concentration in the body, preventing drug accumulation in non-target tissues, and assessing safety and toxicity must be carefully considered, necessitating extensive clinical trials. Finally, therapies involving gene regulation also require ethical evaluations. These challenges and issues call for further experimentation, refinement, and optimization.

In recent years, circRNA vaccines have gained increasing attention. It is undeniable that while the circular structure of circRNA vaccines enhances stability, the processes of circularization and purification remain complex, and residual non-target sequences may introduce unnecessary immunogenicity. This has also driven more researchers to explore simpler synthesis methods. Furthermore, the efficient delivery system for circRNA vaccines still requires optimization to ensure precise delivery to target cells while avoiding degradation. Similarly to ncRNA therapies, circRNA vaccines also require extensive clinical trials to establish dosage standards, assess safety and side effects, and ensure their efficacy and reliability.

Moreover, to promote the application of ncRNAs as biomarkers, therapeutics, and novel vaccines, obtaining regulatory approval is the first step. Additionally, reducing production costs, simplifying drug synthesis methods, and addressing potential adverse reactions must also be considered.

In conclusion, ncRNAs present a promising strategy and significant opportunities for disease treatment but also face inevitable challenges. With more in-depth and valuable research, these issues will eventually be overcome.

## 6. Conclusions

NcRNAs play a crucial role in the regulation of gene expression and have increasingly been studied in recent years as disease biomarkers, innovative therapeutic approaches, and novel vaccines. The exploration of ncRNAs has opened new horizons for understanding disease mechanisms, preventing diseases, and developing treatments. As biomarkers, ncRNAs have demonstrated significant diagnostic and prognostic value in various diseases. With further research, some dysregulated ncRNAs in diseases may mediate changes in target gene expression levels, providing greater insights into disease pathogenesis and aiding in the development of targeted therapies. Concurrently, RNA therapies that regulate gene expression by synthesizing or inhibiting ncRNA molecules offer new possibilities for precise treatments. Furthermore, ncRNAs have shown great potential in eliciting specific immune responses as emerging vaccines.

In spite of the remarkable insights and promises ncRNAs bring, challenges remain. Firstly, accurately measuring and quantifying the expression levels of ncRNAs remains a complex issue, necessitating the development of more sensitive and specific detection methods. Secondly, we need a deeper understanding of the mechanisms of action of different types of ncRNAs in various diseases to ensure their reliability and accuracy. Additionally, ensuring the stability, delivery efficiency, and safety of ncRNA therapeutics is also a critical concern. Despite these challenges, an increasing number of studies and clinical trials indicate that ncRNAs offers more solutions in disease diagnosis, prevention, and treatment, providing new opportunities for the future development of precision medicine.

In conclusion, ncRNAs have revealed promising strategies and immense potential in disease diagnosis and treatment. The future of ncRNAs in medicine is expected to be transformative, heralding advanced, personalized, and effective strategies for managing and eradicating diseases in the coming years.

## Figures and Tables

**Table 2:** Summary of ncRNAs as potential disease biomarkers.

Related Diseases	Biofluid	Potential Biomarkers	Target Genes/Pathways/Mechanistic Approaches	References
Thyroid Cancer	Plasma	miR-21 and miR-181a-5p	N/A	[75]
Liver Cancer	CCM and serum	miR-1247-3p	*B4GALT3*	[76]
Breast Cancer	MSC CCM	miR-21-5p	*S100A6*	[77]
Sepsis	Plasma	miR-1-3p	*SERP1*	[78]
Heart Failure	Plasma	miR-146a	*IRAK-1*, *TRAF6*, *NOX-4* *SMAD4*, and *TGF-β*	[79]
Myocardial Infarction	Cardiac telocyte CCM	miR-21-5p	*CDIP1*	[80]
Alzheimer’s Disease	Plasma	miR-451a and miR-21-5p	N/A	[81]
Depressive Disorder	Cortical neuron CCM	miR-138	*SIRT1*	[82]
Duchenne Muscular Dystrophy	DMD cardiomyocytes CCM	miR-339-5p	*MDM2*, *GSK3A* and *MAP2K3*	[83]
Gout	Plasma	miR-3146	Mediates NETs formation	[84]
Gastric Cancer	Plasma	circ-RanGAP1	*miR-877-3p/VEGFA* axis	[85]
Hepatocellular Carcinoma	HCC CCM	circRNA-100338	N/A	[86]
Glioma	GBM CCM	circNEIL3	Stabilizing *IGF2BP3*	[87]
Myeloma-Related Myocardial Damage	Serum	circ-G042080	*miR-4268/TLR4* axis	[88]
Alzheimer’s Disease	Serum	circ_0003611	*miR-885-5p/KREMEN1* axis	[89]
Type 2 Diabetes	Serum	circGlis3	Regulates *GMEB1* degradation and *HSP27* phosphorylation	[90]
Rheumatoid Arthritis	Plasma	circRNA_09505	*miR-6089/AKT1/NF-κB* axis	[91]
Systemic Lupus Erythematosus	Plasma	hsa_circ_0000479	Metabolic and the Wnt signaling pathway	[92]
Graves’ Disease	Plasma	hsa_circ_0090364	*hsa-miR-378a-3p/IL-6ST/IL21R* axis	[93]
Prostate Cancer	Serum	HOXD-AS1	*miR-361-5p/FOXM1* axis	[94]
Breast Cancer	Serum	SNHG16	*miR-16–5p/SMAD5* axis	[95]
Bladder Cancer	Urine	lncBCYRN1	Activates *WNT5A/VEGF-C/VEGFR3* feedforward loop	[96]
Atherosclerosis	HUVEC CCM	lnc-KCNC3-3:1	*JAK1/STAT3* signaling pathway	[97]
Heart Failure	Plasma	lncRNA-NRF	N/A	[98]
Parkinson’s Disease	Plasma	lnc-MKRN2-42:1	N/A	[99]
Alzheimer’s Disease	Plasma	BACE1-AS	N/A	[100]
Osteoarthritis	OA CCM	SNHG7	*miR-34a-5p/SYVN1* axis	[101]
Crohn’s Disease	Plasma	LUCAT1	NA	[102]
Diabetic Retinopathy in Type 2 Diabetes	Plasma	NR1F1-AS2	Moderates EndMT	[103]

CCM: cell culture media; B4GALT3: beta-1,4-galactosyltransferase 3; MSC: mesenchymal stem cell; S100A6: S100 calcium binding protein A6; SERP1: stress-associated endoplasmic reticulum protein 1; HCC: hepatocellular carcinoma; IRAK-1: interleukin 1 receptor-associated kinase 1; TRAF6: TNF receptor-associated factor 6; NOX-4: NADPH oxidase 4; SMAD4: SMAD family member 4; TGF-β: transforming growth factor beta; CDIP1: cell death-inducing P53 target 1; SIRT1: sirtuin 1; DMD: Duchenne muscular dystrophy; MDM2: MDM2 proto-oncogene; GSK3A: glycogen synthase kinase 3 alpha; MAP2K3: mitogen-activated protein kinase kinase 3; NETs: neutrophil extracellular traps; VEGFA: vascular endothelial growth factor A; GBM: glioblastoma multiforme; IGF2BP3: insulin-like growth factor 2 MRNA binding protein 3; TLR4: toll-like receptor 4; KREMEN1: kringle-containing transmembrane protein 1; GMEB1: glucocorticoid modulatory element binding protein 1; HSP27: heat shock protein 27; AKT1: AKT serine/threonine kinase 1; NF-κB: nuclear factor kappa B; IL-6ST: interleukin 6 cytokine family signal transducer; IL21R: interleukin 21 receptor; FOXM1: forkhead box M1; SMAD5: SMAD family member 5; WNT5A: wnt family member 5A; VEGF-C: vascular endothelial growth factor C; VEGFR3: vascular endothelial growth factor receptor 3; JAK1: Janus kinase 1; STAT3: signal transducer and activator of transcription 3; SYVN1: synoviolin 1; EndMT: endothelial-to-mesenchymal transition.

**Table 3:** RNA therapeutics approved by the FDA and/or EMA.

Drugs	Types	Route of Administration	Target Organ	Mechanism of Action	Related Diseases	FDA and/or EMA Approval Year	References
Fomivirsen	ASO	IVT	Eye	Targeting and silencing the mRNA of CMV IE2 protein	CMV retinitis in immunocompromised patients	FDA (1998) EMA (1999)	[104]
Pegaptanib	Phosphate oligonucleotide aptamer	IVT	Eye	Inhibiting VEGF-165	nAMD	FDA (2004)	[111]
Mipomersen	ASO	SC	Liver	Targeting and silencing the mRNA of apolipoprotein B to reduce LDL levels	HoFH	FDA (2013) EMA (2012)	[112]
Eteplirsen	ASO	IV	Muscle	Targeting and splicing the pre-mRNA of defective DMD dystrophin protein	DMD	FDA (2016)	[113]
Nusinersen	ASO	ITH	Central nervous system	Targeting and splicing the pre-mRNA of defective SMN protein	SMA	FDA (2016) EMA (2017)	[114]
Patisiran	siRNA	IV	Liver	Targeting and silencing the mRNA of TTR protein to prevent the production of TTR protein	ATTR amyloidosis	FDA (2018)	[106]
Inotersen	ASO	SC	Liver	Targeting and silencing the mRNA of TTR protein to prevent the production of TTR protein	ATTR amyloidosis	FDA (2018) EMA (2018)	[115]
Givosiran	siRNA	SC	Liver	Targeting and silencing the mRNA of ALAS1 to reduce ALAS1 levels	AHP	FDA (2019) EMA (2020)	[116]
Golodirsen	ASO	IV	Muscle	Targeting the splicing of DMD pre-mRNA (exon 53 skipping)	DMD	FDA (2019)	[117]
Volanesorsen	ASO	SC	Liver	Targeting and silencing the mRNA of APOC3 to reduce triglyceride production	FCS	EMA (2019)	[118]
Viltolarsen	ASO	IV	Muscle	Targeting the splicing of DMD pre-mRNA (exon 53 skipping)	DMD	FDA (2020)	[119]
Lumasiran	siRNA	SC	Liver	Targeting and silencing the mRNA of HAO1 to reduce GO levels	PH1	FDA (2020) EMA (2020)	[120]
Casimersen	ASO	IV	Muscle	Targeting the splicing of DMD pre-mRNA (exon 45 skipping)	DMD	FDA (2021)	[121]
Inclisiran	siRNA	SC	Liver	Targeting PCSK9 to inhibit its synthesis and reduce LDL-C levels	ASCVD	FDA (2021) EMA (2020)	[122]
Vutrisiran	siRNA	SC	Liver	Targeting and silencing the mRNA of TTR protein to prevent the production of TTR protein	ATTR amyloidosis	FDA (2022) EMA (2022)	[123]
Nedosiran	siRNA	SC	Liver	Targeting liver LDH mRNA to reduce the expression of LDH	PH1	FDA (2023)	[124]
Eplontersen	ASO	SC	Liver	Targeting and silencing the mRNA of TTR protein to prevent the production of TTR protein	ATTR amyloidosis	FDA (2023)	[125]
Tofersen	ASO	ITH	Muscle	Targeting SOD1 mRNA to reduce the synthesis of SOD1 protein	SOD1-ALS	FDA (2023) EMA (2023)	[126]
Olezarsen	ASO	SC	Liver	Reducing hepatic synthesis of apolipoprotein C-III to lower plasma triglyceride levels	FCS	FDA (2024)	[127]

ASO: antisense oligonucleotide; siRNA: small interfering RNA; IVT: intravitreal; SC: subcutaneous; IV: intravenous; ITH: intrathecal; CMV: cytomegalovirus; IE2: immediate Early 2; VEGF-165: vascular endothelial growth factor 165; LDL: low-density lipoprotein; DMD: Duchenne muscular dystrophy; SMN: survival motor neuron; TTR: transthyretin; ALAS1: 5′-aminolevulinate synthase 1; APOC3: apolipoprotein CIII; HAO1: hydroxyacid oxidase 1; GO: glycolate oxidase; PCSK9: proprotein convertase subtilisin/kexin Type 9; LDL-C: low-density lipoprotein cholesterol; nAMD: neovascular age-related macular degeneration; HoFH: homozygous familial hypercholesterolemia; SMA: spinal muscular atrophy; ATTR: transthyretin amyloidosis; AHP: acute hepatic porphyria; ASCVD: atherosclerotic cardiovascular disease; PH1: primary hyperoxaluria type 1; SOD1-ALS: amyotrophic lateral sclerosis (ALS) associated with a mutation in the superoxide dismutase 1 (SOD1) gene; FCS: familial chylomicronemia syndrome.

**Table 4:** Summary of circRNA vaccines.

Vaccines	Cyclization Strategy	Delivery	Antigen	References
circRNA^RBD^	Ribozymatic autocatalysis	LNP	SARS-CoV-2 RBD antigen	[149]
VFLIP-X	T4 RNA ligase	LNP	SARS-CoV-2 spiking protein	[151]
circRNA^OVA-luc^-LNP	Ribozymatic autocatalysis	LNP	OVA [257-264]-luciferase	[150]
CircRNA encoding cytokines	Ribozymatic autocatalysis	LNP	Active IL-15\IL-12\GM-CSF\IFN-α 2b	[152]
cirA29L, cirA35R, cirB6R, and cirM1R	Ribozymatic autocatalysis	LNP	MPXV proteins A29L, A35R, B6R, and M1R	[153]
circRNA3×PTPN2	Ribozymatic autocatalysis	LNP	PTPN2	[154]
circRNA-G	Ribozymatic autocatalysis	LNP	Glycoproteins of the RABV vaccine strain SAD-L16	[155]
IL12-circRNA	Ribozymatic autocatalysis	LNP	IL-12	[156]
circRNA-NA	Ribozymatic autocatalysis	LNP	NA	[157]
EDIII-Fc circRNA	Ribozymatic autocatalysis	LNP	Dimeric EDIII-Fc fusion	[158]

RBD: receptor-binding domain; SARS: severe acute respiratory syndrome; CoV: coronavirus; OVA: ovalbumin; IL: interleukin; GM-CSF: granulocyte-macrophage colony-stimulating factor; IFN: interferon; MPXV: monkeypox virus; PTPN2: protein Tyrosine Phosphatase Non-Receptor Type 2; RABV: rabies virus; NA: multi-subtype neuraminidases; EDIII: domain III of Zika virus envelope protein.
